# Role of *Daucus carota* in Enhancing Antiulcer Profile of Pantoprazole in Experimental Animals

**DOI:** 10.3390/molecules25225287

**Published:** 2020-11-13

**Authors:** Syed Mohammed Basheeruddin Asdaq, Earla Swathi, Sunil S Dhamanigi, Mohammed Asad, Yahya Ali Mohzari, Ahmed A. Alrashed, Abdulrahman S. Alotaibi, Batool Mohammed Alhassan, Sreeharsha Nagaraja

**Affiliations:** 1 Department of Pharmacology, College of Pharmacy, AlMaarefa University, Riyadh 13713, Saudi Arabia; 2 Department of Pharmacology, Krupanidhi College of Pharmacy, Bangalore 560035, India; emadfaiqa@gmail.com (E.S.); qualityasdaq@gmail.com (S.S.D.); 3 College of Applied Medical Sciences, Shaqra University, Shaqra 11911, Saudi Arabia; basheer_1@rediffmail.com; 4 Clinical Pharmacy Department, King Saud Medical City, Riyadh 12746, Saudi Arabia; yali2016@hotmail.com; 5 Pharmaceutical Service Department, Inpatient Pharmacy, King Fahad Medical City, Riyadh 11525, Saudi Arabia; alarashed@gmail.com (A.A.A.); mhospital1920@gmail.com (A.S.A.); 6 Neonatal Intensive Care Unit, AlMoosa Specialist Hospital, Riyadh 36342, Saudi Arabia; batool42@gmail.com; 7 Department of Pharmaceutical Sciences, College of Clinical Pharmacy, King Faisal University, Al-Ahsa 31982, Saudi Arabia; sharsha@kfu.edu.sa; 8 Department of Pharmaceutics, Vidya Siri College of Pharmacy, Off Sarjapura Road, Bangalore 560035, India

**Keywords:** duodenal ulcer, gastric acid, gastric cytoprotection, gastric secretion, mucin

## Abstract

The carrot plant *(Daucus carota*) and its components are traditionally reported for the management of gastric ulcers. This study was performed to evaluate the role of carrot when administered concurrently with a conventional antiulcer treatment, pantoprazole, in alleviating gastric and duodenal ulcers in female experimental animals. The study involved standard animal models to determine the ulcer preventive effect using pylorus ligation, ethanol, and stress induced acute gastric ulcer models and duodenal ulcer models involving cysteamine. Acetic acid-induced chronic gastric ulcer and indomethacin-induced gastric ulcer models were used to evaluate the ulcer healing effect. Carrot fruit (500 mg/kg) and its co-administration with pantoprazole produced significant protection in an ethanol- and stress-induced acute gastric ulcer and cysteamine-induced duodenal ulcer. The healing of the acetic acid-induced chronic gastric ulcer was also augmented with this combination. Both total proteins and mucin contents were significantly increased in indomethacin-induced gastric ulcers. Similarly, in pylorus ligation, the pepsin content of gastric juice, total acidity, and free acidity were reduced. Overall, both ulcer preventive effects and ulcer healing properties of the pantoprazole were significantly enhanced in animals who received the co-administration of carrot fruit (500 mg/kg).

## 1. Introduction

A diet enriched with fruits and vegetables obviates gastrointestinal manifestations, including the prevention of new gastric or duodenal ulcers and healing of already formed ulcers [1]. Carrot (*Daucus carota* L) belongs to the family Apiaceae. The edible part of a carrot is a taproot that gets its unique color due to the presence of β-carotene that is metabolized into vitamin A after human consumption. Carrot fruits are reported to cleanse the intestines as well as act as a diuretic. They are the source of nutrition and help in maintaining an acid–base balance. They are regarded as commonly used vegetables for vision maintenance. The beneficial effect of carrot and its active constituent, β-carotene, is also reported for liver function. Additionally, carrots provide relief from diarrhea, constipation, intestinal inflammation, weakness, illness, and in the treatment of rickets. In addition to β-carotene, the protective actions of carrot are also attributed to its other constituents, such as riboflavin, A-retinol, niacin, A-carotenoid, vitamins A, B_6_, B_12_, C, E, and K, thiamin, pantothenic acid, and folate [2].

Although the concurrent use of herbal therapies and home remedies, along with conventional modern medicinal products, is commonly found for the management of many diseases and ailments, there is a need to standardize their combined use to overcome the possible herb–drug interaction. Their concomitant use may either decrease or increase the therapeutic value of one another [3].

Even though carrot fruit is employed in a number of traditional medicinal systems in India for a variety of ailments in the gastrointestinal system, so far, there is no scientific report to confirm the ethnopharmacological claim. We also felt it necessary to determine the biological role of carrot in the presence of conventional antiulcer drugs, such as potent acid suppressor agent, proton pump inhibitor (PPI), and pantoprazole, as many patients use carrot with these drugs. Although PPI is a potent antiulcer agent, overdependence on this class of agent may lead to an increased risk of bone fracture [4], mineral and vitamin deficiencies [5], and *Clostridium difficile* infection [6]. Additionally, direct correlation is reported between the use of PPIs and development of pneumonia [7], dementia [8], gastric cancer [9], and chronic kidney disease [10]. This has led the FDA to release a number of safety guidelines and publish recommendations for PPI usage [11]. Thus, there is a need to explore additives or substitutes that can complement or supplement the use of PPIs. Therefore, this study was carried out to validate the traditional gastroprotective claim of carrot and also to determine its interaction with pantoprazole (PZL) for antiulcer activity using different experimental models.

## 2. Results

### 2.1. Phytochemical Investigation

Preliminary phytochemical investigation of the carrot suspension confirmed the presence of carotenoids and phenolic compounds. The presence of carbohydrates, triterpenoids glycosides, saponins, tannins, alkaloids, steroids, proteins, amino acids, and flavonoids were also observed (Table S1).

### 2.2. Antioxidant Effect of Carrot

Using DPPH method, gallic acid exhibited in-vitro antioxidant activity at IC_50_ values of 6 µg/mL, while carrot showed at 312 µg/mL. IC_50_ is the concentration of an inhibitor at which 50% inhibition of the response is seen (Table 1).

### 2.3. Acetic Acid Induced Gastric Ulcers

As expected, pantoprazole increased the healing of ulcers. However, carrot showed an effect only at the higher dose of 500 mg/kg. The combined administration of high dose of carrot (500 mg/kg) with pantoprazole showed a significant increase in ulcer healing compared to pantoprazole administered alone, though such an effect was not observed with the combined use of low dose of carrot (200 mg/kg) with pantoprazole (Figure S1). Ulcer healing was determined by a fall in ulcer score, decrease in ulcer index with an increased collagen content volume, rise in capillary density, enhancement in surface epithelium, and the regeneration of glandular epithelium width (Table 2).

### 2.4. Pylorus Ligated Rats

The gastric antisecretory as well as antiulcer effect of carrot was observed only in higher dose (500 mg/kg) in pylorus ligated rats. However, the lower dose (200 mg/kg) of carrot reduced free acidity and total acidity of the gastric juice. The use of pantoprazole alone or in combination with high dose of carrot (500 mg/kg) showed excellent effects (Figure S2). Moreover, combined therapy of carrot at higher dose and pantoprazole produced a better result than when pantoprazole was given alone (Table 3).

### 2.5. Ethanol and Stress Induced Ulcers

As shown in Table 4 and Table 5, the administration of carrot at high dose (500 mg/kg), pantoprazole, as well as their combination prevented ulcer formation in both ethanol- (Figure S3) and stress-(Figure S4) induced gastric ulcer animal experimental models. The combined therapy of high dose of carrot with pantoprazole was more effective compared to single drug treatment.

### 2.6. Cysteamine Induced Duodenal Ulcers

Table 6 shows a significant reduction in ulcer area in all treated groups while the lower dose (200 mg/kg) of carrot did not significantly affect the ulcer score, though all other treatments produced a significant fall in ulcer score. Significantly, a high level of effect was noticed in groups which received high dose of carrot (500 mg/kg) and pantoprazole when compared to the individual treatment of drugs (Figure S5).

### 2.7. Indomethacin Induced Gastric Ulcers

Similar to earlier effect on ulcers, high dose of carrot (500 mg/kg), pantoprazole and their combination reduced ulcer index and increased gastric mucin content in animals with gastric ulcers induced by indomethacin (Figure S6). Furthermore, carrot at both doses and their combination with pantoprazole showed good antioxidant effect, as indicated by an increase in superoxide dismutase activity (Table 7).

## 3. Discussion

The present work showed gastric antisecretory and gastric cytoprotective effects of *Daucus carota* fruit and its additive effect with the antiulcer drug pantoprazole. The *Daucus carota* plant is traditionally used as an antiulcer agent in several regions of the world [12]. An earlier study showed that the methanolic and aqueous extract of carrot plant was found to possess a cytoprotective effect in the gastric region against gastric ulcers in rats induced by ethanol [13]. The current study was carried out using only fruits that are traditionally used in India and the results confirmed its antiulcer effect. Furthermore, the additive effect observed with the combination of carrot and antiulcer agent suggests that it is not only safe to consume carrot fruit along with antiulcer effects, but an enhanced therapeutic effect may be achieved with these combinations.

The standard antiulcer methods used for the evaluation of antiulcer activity were followed. Only female rats of reproductive age used to minimize hormone mediated bias that would have occurred in case of mixed gender. It is observed that the ratio of men to premenopausal women for ulcer development is high, ranging from 1.9:1 to 3.1:1 [14]. The acetic acid-induced gastric ulcer model was used to evaluate healing of gastric ulcers, wherein both gastric cytoprotective and gastric antisecretory agents as well as antioxidants are reported to increase gastric ulcer healing [15]. Additionally, since carrot was reported for its cytoprotective effect [16], models involving the induction of gastric ulcers by indomethacin and ethanol were used to determine the cytoprotective action of carrot and its combination. Animal models involving mental stress through cold plus restraint and pyloric ligation evaluated the gastric ulcer preventive effect. The best method used for the development of duodenal ulcers is the oral administration of cysteamine. The mechanism of induction of ulcers through the above-mentioned chemicals/methods has been described by us earlier [17].

Carrot is used traditionally in different forms. Scientific studies on its pharmacological effects have been reported using different extracts, ranging from polar aqueous extract to non-polar solvents [18,19]. Some reports suggest that boiled carrot is more effective than raw carrot [12]. We used boiled extract in this study.

Carrot is known to contain several constituents that include phenolics, flavonoids, and volatile oils. It is a rich source of β-carotene and pectins [20]. Phenolics and flavonoids present in carrot are reported for their antioxidant properties [21]. Moreover, carrot is known to contain C17-polyacetylenes such as falcarinol, which are a group of oxylipins that are known for anti-inflammatory, anti-neoplastic, antibacterial, serotonergic, and antifungal properties [22]. Since falcarinol is widely believed to improve human health and protect against a variety of diseases, studies concerning its contribution to the antiulcer effect of carrot may help in identifying other plants containing this chemical for potential antiulcer actions. Volatile oils present in carrots have also been reported to possess a number of activities. Around 34 volatile constituents that include major chemicals such as carotol, β-bisabolene, and isoelemicin are reported in carrot [23].

Since carrot contains a wide variety of constituents with varying effects, it can be assumed that one single constituent may not be responsible for its antiulcer action. Furthermore, carrot prevented the development of ulcers and increased ulcer healing by more than one mechanism, including gastric antisecretory, gastric cytoprotective, and antioxidant effects, as shown in different models used in this study. It is possible that each of the ulcer healing and ulcer preventive effects may be due to one or more constituents of the carrot preparation. We would like to emphasize that this study was not meant to identify or isolate chemical constituents responsible for the antiulcer effect, but rather to verify the traditional claim that carrot fruit possesses antiulcer action. Moreover, it is very unlikely that carrot or its isolated constituents may be used alone as antiulcer drugs, as several traditional medicines having a more potent effect than carrot have at lower dose and some of them are being used. The low dose of carrot (200 mg/kg) did not show cytoprotective potential or a weak effect in ulcer models. Hence, the effect of a combination of carrot with pantoprazole (proton pump inhibitor) was evaluated. The additive effect seen with this combination could be due to the combined antiulcer action of each agent and/or there may be some pharmacokinetic interactions. Further studies on pharmacokinetics may provide information about the potential interaction between carrot and these drugs.

## 4. Materials and Methods

### 4.1. Experimental Animals

Institutionally bred albino Wistar rats (female, three to four months old) in the weight ranging between 150 and 200 g were used in this study. The Institutional ethical committee approved the experimental protocol of this study with the number KCP/IAEC-22/2008-09 dated 1 December 2008. Standard conditions were followed before and during the experimentation for the maintenance of animals as proposed by Committee for the Purpose of Control, and Supervision on Experiments on Animals (CPCSEA).

### 4.2. Method to Prepare Carrot Solution and Selection of Dose

The suspension of Carrot (*Daucus carota*) fruit was prepared by steam boiling of carrot. Light cooking is known to increase its antioxidant property [24]. Carrot has more nutrition in cooked rather than raw form. After boiling, it was triturated using a mortar and pestle by adding water (1 g/2 mL). Addition of water enabled easy administration. Since carrot is a widely used in large quantities, toxicity study was not done to explore the dose to be used. Two doses were selected based on the traditional and local healers claim for a possible therapeutic effect for gastric disturbances, namely 200 mg/kg body weight (low dose) and 500 mg/kg body weight (high dose). The rat equivalence dose was calculated based on recommended human dose (1.5 to 2 g/kg body weight for human consumption-around 100 g carrot per day for an adult weighing 60–65 kg) using a reference table given by Nair and Jacob [25].

### 4.3. Phytochemical Studies

The phytochemical analysis of aqueous suspension of boiled carrot fruit *(Daucus carota)* was done to determine the presence of phytochemicals, like tannins, phytosterols, carbohydrates, flavonoids, proteins, glycosides, and saponins [26]. Hager’s, Mayer’s, Dragendroff’s, and Wagner’s tests were used to check the presence of alkaloids, whereas Molisch, Felhing’s, Barfoed’s, and Benedictt’s tests were employed for detecting carbohydrates. The presence of proteins in the extract were determined by Millon’s, Biuret, and Ninhyrin tests. Further, Salkowski, Liebermann–Burchard, Baljet, and Keller–Killani’s tests were used for determining steroids, triterpenoids, and cardiac glycosides. Finally, ferric chloride and lead Acetate tests were performed to assess the presence of tannins and flavonoids.

### 4.4. Antioxidant Test (DPPH Free Radical Scavenging Activity)

An adequate amount of carrot was dissolved in 98% methanol to prepare stock of the aqueous solution of carrot (10 mg/mL). Using an appropriate dilution from the stock solution, working solutions of 10, 20, 30, 40, 80, 100, 120, 140, 180, 200, 250, 400, and 800 μg/mL were prepared. The antioxidant potential of carrot solution and gallic acid (standard) was determined on 1, 1-diphenyl-2-picrylhydrazyl (DPPH) [27].

### 4.5. Grouping of Experimental Animals and Animal Models

The experimental rats were divided into six groups. Group I received a vehicle (1 mL/kg), while group II and III were given low (LCE, 200 mg/kg) and high (HCE, 500 mg/kg) doses of carrot solution. The animals of group IV were administered with pantoprazole (20 mg/kg) [28] (PZL), whereas V and VI groups were treated with LCE plus PZL and HCE plus PZL, respectively.

#### 4.5.1. Acetic Acid Induced Chronic Gastric Ulcers

At the end of 10 days treatment of animals, as per the protocol of the group described above, animals were kept under fasting conditions for 24 h and the abdomen was opened under light ether anesthesia [29]. A cylindrical mould of 6 mm in diameter was used to cover the serosal surface of the stomach and 0.05 mL of the glacial acetic acid (0.05 mL) was poured via this mould for about 60 s. The computer scanner was used to scan the stomach samples. Both mucosal area and total ulcerated area were measured based on a method developed by National Institute of Health, USA using a public domain image processing and analysis program. The free desktop version was downloaded from Scion (http://www.scioncorp.com). The ulcer index was calculated [30]. The ulcer index was determined using the formula Ulcer index = 10/X, where X = Total mucosal area/Total ulcerated area. The ulcers were given scores based on their intensity as 0 = no ulcer, 1 = superficial mucosal erosion, 2 = deep ulcer or transmural necrosis, 3 = perforated or penetrated ulcer. Subsequently, stomach samples were processed for histological investigation and capillary density, collagen content, regenerated lining epithelial width as well as glandular epithelial width were studied to evaluate the rate and extent of healing of ulcer [31].

#### 4.5.2. Pylorus Ligation Induced Ulcers

Experimental animals were kept for 36 h fasting prior to pyloric ligation with water *ad libitum*. Ether anesthesia was used to anesthetized animals and pyloric sphincter was ligated, drugs as per the experimental group were administered intraduodenally subsequent to the ligation of pylorus. No food or water were offered to animals during the post-surgery period. Animals were sacrificed 6 h after pyloric ligation [32]. Gastric juice was collected from the stomach to estimate both free acidity and total acidity, as well as pepsin, total proteins, mucin contents [29]. Additionally, the ulcer score and ulcer index were calculated as per the method described above.

#### 4.5.3. Ethanol Induced Ulcers

Ethanol was administered to experimental animals after keeping them under fasting conditions for 36 h. Drug was administered to the animals based on the group protocol described above 1 h prior and post ethanol administration. Subsequently, animals were sacrificed, and the stomach was isolated. Then, both ulcer score and ulcer index were calculated based on the method described elsewhere [33].

#### 4.5.4. Cold Restraint Stress Induced Ulcers

Animals were administered with drugs based on the experimental group 30 min before stress induction. Stress was induced by keeping them in a restraint cage for 3 h at 2 °C for 3 h. At the end of 3 h, stomachs were isolated after sacrificing animals. Then, the ulcer score and ulcer index were calculated [34].

#### 4.5.5. Healing of Indomethacin-Induced Gastric Ulcers

All experimental animals used in this model were administered with indomethacin 5 mg/kg orally for five days. Indomethacin solution was prepared daily with sodium carbonate adjusted to pH 7.4 with HCl. Subsequently, they are treated with their respective drug regimen for five days, animals were sacrificed [35]. Both ulcer score and ulcer index were calculated. Additionally, a glandular part of the stomach was used to estimate the presence of superoxide dismutase activity (SOD), mucin, and total protein contents [29].

#### 4.5.6. Cysteamine Induced Duodenal Ulcers

Experimental animals received drugs as per their group protocol 30 min before the dose of cysteamine hydrochloride (400 mg/kg) orally. The second dose of drug was administered and so the second dose of cysteamine on the same day after an interval of four h [36]. Animals were sacrificed at the end of 24 h following the last dose of cysteamine. The duodena were cut open along the antimesenteric side. The ulcerated area in the duodenum, ulcer score, and ulcer index were determined using the method described earlier [37].

### 4.6. Statistical Analysis

One-way analysis of variance (ANOVA) followed by Dunnett’s comparison test was used to determine the statistical significance of interventions in the study. All values are expressed as mean ± SEM and *p* < 0.05 was considered significant.

## 5. Conclusions

To conclude, *Daucus carota* fruit and its combination with pantoprazole obviate the formation of gastric and duodenal ulcers and enhance gastric ulcer healing. The antioxidant profile of carrot together with the gastric antisecretory properties of some of the active constituents of carrot preparation and probable gastric cytoprotection offered by vitamin A or beta-carotene may be responsible for its antiulcer effect. Overall, both ulcer preventive effects and ulcer healing properties of the pantoprazole were significantly enhanced in animals who received the co-administration of carrot fruit (500 mg/kg).

## Figures and Tables

**Table 1:** Antioxidant activity of *Daucus carota.*

Sl.No	Test Compound	IC_50_ (µg/mL)	Weight of the Extract (g %, *w*/*v*)
1.	Gallic acid	6	1
2.	Carrot fruit (*Daucus carota*)	312	1

**Table 2:** Impact on gastric histological profile in acetic acid induced animal model.

Groups	CD	VCC	SE	UI	RGE	US
Control	4.30 ± 0.3	0.06 ± 0.0	133.30 ± 0.8	1.16 ± 0.0	389 ± 28.5	21.33 ± 1.8
PZL	6.30 ± 0.3 *	0.10 ± 0.0 **	155.60 ± 1.2 **	0.74 ± 0.0 **	499 ± 0.5 **	12.33 ± 0.8 **
LCE	4.30 ± 0.3	0.06 ± 0.0	133.20 ± 0.8	1.06 ± 0.0	361 ± 18.7	14.33 ± 1.4 *
HCE	6.30 ± 0.3 *	0.08 ± 0.0 *	111.60 ± 3.3 **	0.40± 0.0 ***	389 ± 28.5	12.66 ± 1.3 **
LCE + PZL	6.30 ± 0.3 *	0.09 ± 0.0 **	163.00 ± 1.5 ***	0.42 ±0.0 ***^,†^	506 ± 7.0 **	5.33 ± 1.4 ***^,†^
HCE + PZL	7.00 ± 0.5 **^,†^	0.10 ± 0.0 **	173.60 ± 2.0 ***^,†^	0.33 ± 0.0 ***^,††^	510 ± 6.9 **^,†^	3.00 ± 0.5 ***^,††^

Data presented as Mean ± SEM, * *p* < 0.1, ** *p* < 0.01, *** *p* < 0.001 compared with control; ^†^ *p* < 0.1, ^††^ *p* < 0.01 compared with PZL; PZL (Pantoprazole 20 mg/kg, orally); LCE (Low dose carrot 200 mg/kg, orally), HCE (High dose carrot 500 mg/kg, orally); US (Ulcer score); UI (Ulcer index); SE (surface epithelium- µm); VCC (volume of collagen content); CD (Capillary density (No) in 19,600 µm^2^); RGE (Regenerated glandular epithelium width-µm).

**Table 3:** Impact on gastric histological profile in pylorus ligated rats.

Groups	Ulcer Index	Ulcer Score	Free Acidity (mEq/L)	Total Acidity (mEq/L)	Mucin Content (μg/g)	Pepsin Content (μg/6 h)	Total Protein (mg/mL)
Control	0.37 ± 0.0	17.00 ± 1.	45.50 ± 3.8	56.20 ± 5.8	11.20 ± 0.9	0.19 ± 0.0	44.95 ± 2.5
PZL	0.25 ± 0.0 *	9 ± 0.5 **	5.00 ± 1.0 **	10.00 ± 0.9 **	30.57 ± 2.6 *	0.11 ± 0.0 ***	30.30 ± 1.9 **
LCE	0.27 ± 0.0	12.33 ± 1.4	21.00 ± 1.7 **	36.20 ± 2.4 *	24.68 ± 2.9	0.18 ± 0.0	40.80 ± 1.7
HCE	0.15 ± 0.0 ***	7.30 ± 1.7 ***	14.00 ± 3.1**	20.00 ± 2.1 **	38.3 ± 5.3 **	0.13 ± 0.0 **	27.31 ± 0.8 **
LCE + PZL	0.24 ± 0.0 *	2.33 ± 0.3 ***^,†^	2.20 ± 1.0 ***^,†^	5.00 ± 0.4 ***^,†^	56.60 ± 6.2 ***^,††^	0.06 ± 0.0 ***^,††^	15.90 ± 2.6 ***^,††^
HCE + PZL	0.11 ± 0.0 ***^,††^	0.60 ± 0.6 ***^,††^	0.90 ± 0.0 ***^,†^	1.20 ± 0.0 ***^,†^	78.06 ± 2.7 ***^,†††^	0.02 ± 0.0 ***^,†††^	14.00 ± 1.1 ***^,††^

Data given as Mean ± SEM (*n* = 6), * *p* < 0.1, ** *p* < 0.01, *** *p* < 0.001 compared with control; ^†^ *p* < 0.1, ^††^ *p* < 0.01, ^†††^ *p* < 0.001 compared with PZL; PZL (Pantoprazole 20 mg/kg, orally); LCE (Low dose carrot 200 mg/kg, orally), HCE (High dose carrot 500 mg/kg, orally.

**Table 4:** Impact on ulcer index and ulcer score in ethanol induced gastric ulcers.

Treatment	Ulcer Index	Ulcer Score
Control	2.03 ± 0.3	16.30 ± 1.3
PZL	0.81 ± 0.0 **	7.00 ± 1.1 ***
LCE	1.36 ± 0.3	13.60 ± 0.3
HCE	1.01 ± 0.0 ***	11.00 ± 0.5 **
LCE + PZL	0.92 ± 0.0 ***^,†^	7.60 ± 0.8 ***
HCE + PZL	0.01 ± 0.0 ***^,†^	2.60 ± 0.3 ***^,†^

Data given as Mean ± SEM (*n* = 6), ** *p* < 0.01, *** *p* < 0.001 compared with control; ^†^ *p* < 0.1 compared with PZL; PZL (Pantoprazole 20 mg/kg, orally); LCE (Low dose carrot 200 mg/kg, orally), HCE (High dose carrot 500 mg/kg, orally).

**Table 5:** Impact on ulcer index and ulcer score in stress induced gastric ulcers.

Treatment	Ulcer Index	Ulcer Score
Control	0.97 ± 0.0	14.00 ± 1.1
PZL	0.35 ± 0.0 ***	6.60 ± 0.8 **
LCE	0.72 ± 0.0	6.60 ± 2.4 **
HCE	0.45 ± 0.0 ***	5.30 ± 0.3 ***
LCE + PZL	0.57 ± 0.0 **	1.30 ± 0.3 ***^,†^
HCE + PZL	0.06 ± 0.0 ***^,†^	0.30 ± 0.3 ***^,††^

Data given as Mean ± SEM (*n* = 6), ** *p* < 0.01, *** *p* < 0.001 compared with control; ^†^ *p* < 0.1, ^††^ *p* < 0.01 compared with PZL; PZL (Pantoprazole 20 mg/kg, orally); LCE (Low dose carrot 200 mg/kg, orally), HCE (High dose carrot 500 mg/kg, orally).

**Table 6:** Impact on ulcer area, ulcer score and ulcer index in cysteamine induced duodenal ulcers.

Treatment	Ulcer Area	Ulcer Index	Ulcer Score
Control	10.60 ± 1.7	2.40	11.00 ± 1.5
PZL	5.00 ± 0.5 **	1.48	6.00 ± 0.5 **
LCE	6.30 ± 0.8 *	2.24	7.33 ± 0.6
HCE	1.00 ± 1.0 ***	0.71	5.66 ± 0.3 **
LCE + PZL	4.60 ± 0.8 **	1.49	5.66 ± 0.8 **
HCE + PZL	0.60 ± 0.3 ***^,†^	0.73	1.00 ± 0.5 ***^,††^

Data given as Mean ± SEM (*n* = 6), * *p* < 0.1, ** *p* < 0.01, *** *p* < 0.001 compared with control; ^†^ *p* < 0.1, ^††^ *p* < 0.01 compared with PZL; PZL (Pantoprazole 20 mg/kg, orally); LCE (Low dose carrot 200 mg/kg, orally), HCE (High dose carrot 500 mg/kg, orally).

**Table 7:** Impact on mucin content, ulcer index, ulcer score, total proteins, anti-oxidant factors in indomethacin induced ulcers.

Treatment	Mucin Content (µg/g)	Ulcer Index	Ulcer Score	Total Proteins (mg/mL)	SOD (Units/mg of Proteins)
Control	0.50 ± 0.0	0.76 ± 0.1	12.66 ± 2.4	19.63 ± 0.5	2.70 ± 0.2
PZL	1.50 ± 0.1 **	0.36 ± 0.0 **	6.00 ± 1.0 **	30.39 ± 1.6 **	5.49 ± 0.9 **
LCE	1.00 ± 0.0	0.74 ± 0.0	6.00 ± 0.5 **	22.20 ± 1.6	2.60 ± 0.3 *
HCE	1.10 ± 0.7 *	0.15 ± 0.0 ***	3.33 ± 0.6 ***	34.04 ± 1.6 ***	4.00 ± 0.3 **
LCE + PZL	1.60 ± 0.3 **	0.04 ± 0.0 ***^,†^	0.66 ± 0.3 ***^,†^	38.75 ± 0.3 ***^,†^	9.10 ± 0.2 ***
HCE + PZL	2.00 ± 0.5 ***^,†^	0.01 ± 0.0 ***^,†^	0.33 ± 0.3 ***^,†^	39.03 ± 2.60 ***^,††^	13.60 ± 0.7 ***^,†^

Data given as Mean ± SEM (*n* = 6), * *p* < 0.1, ** *p* < 0.01, *** *p* < 0.001 compared with control; ^†^ *p* < 0.1, ^††^ *p* < 0.01 compared with PZL; PZL (Pantoprazole 20 mg/kg, orally); LCE (Low dose carrot 200 mg/kg, orally), HCE (High dose carrot 500 mg/kg, orally).

## Supplementary Materials

The following are available online, Figure S1: Photographs showing effect on ulcer healing in acetic acid induced chronic gastric ulcers, Figure S2. Photographs showing effect in pyloric ligation induced gastric ulcer, Figure S3. Photographs showing effect in ethanol induced gastric ulcers, Figure S4. Photographs showing effect in stress induced gastric ulcers, Figure S5. Photographs showing effect in cysteamine induced duodenal ulcers, Figure S6: Photographs showing effect in indomethacin induced gastric ulcers, Table S1: Phytochemical investigation for various compounds.
