# Supplementary material for: Role of Daucus carota in Enhancing Antiulcer Profile of Pantoprazole in Experimental Animals
Source: Molecules. 2020 Nov 13;25(22):5287. doi: 10.3390/molecules25225287 (PMC7696376; doi:10.3390/molecules25225287)
Supplement: Supplementary file 1 [file molecules-25-05287-s001.pdf]

Article

Role of *Daucus Carota* in Enhancing Antiulcer Profile of Pantoprazole in Experimental Animals

Syed Mohammed Basheeruddin Asdaq [1,*], Earla Swathi [2], Sunil S Dhamanigi [2], Mohammed Asad [3], Yahya Ali Mohzari [4], Ahmed A. Alrashed [5], Abdulrahman S. Alotaibi [5], Batool Mohammed Alhassan [6] and Sreeharsha Nagaraja [7,8]

1 Department of Pharmacology, College of Pharmacy, AlMaarefa University, Riyadh 13713, Saudi Arabia
2 Department of Pharmacology, Krupanidhi College of Pharmacy, Bangalore 560035, India; emadfaiqa@gmail.com (E.S.); qualityasdaq@gmail.com (S.S.D.)
3 College of Applied Medical Sciences, Shaqra University, Shaqra 11911 and Saudi Arabia; basheer_1@rediffmail.com
4 Clinical Pharmacy Department, King Saud Medical City, Riyadh 12746, Saudi Arabia; yali2016@hotmail.com
5 Pharmaceutical Service Department, Inpatient Pharmacy, King Fahad Medical City, Riyadh 11525, Saudi Arabia; alarashed@gmail.com (A.A.A.); mhospital1920@gmail.com (A.S.A.)
6 Neonatal Intensive Care Unit, AlMoosa Specialist Hospital, Riyadh 36342, Saudi Arabia; batool42@gmail.com
7 Department of Pharmaceutical Sciences, College of Clinical Pharmacy, King Faisal University, Al-Ahsa, 31982, Saudi Arabia; sharsha@kfu.edu.sa
8 Department of Pharmaceutics, Vidya Siri College of Pharmacy, Off Sarjapura Road, Bangalore 560035, India
* Correspondence: sasdaq@gmail.com; Tel.: +966-1-403555-3399

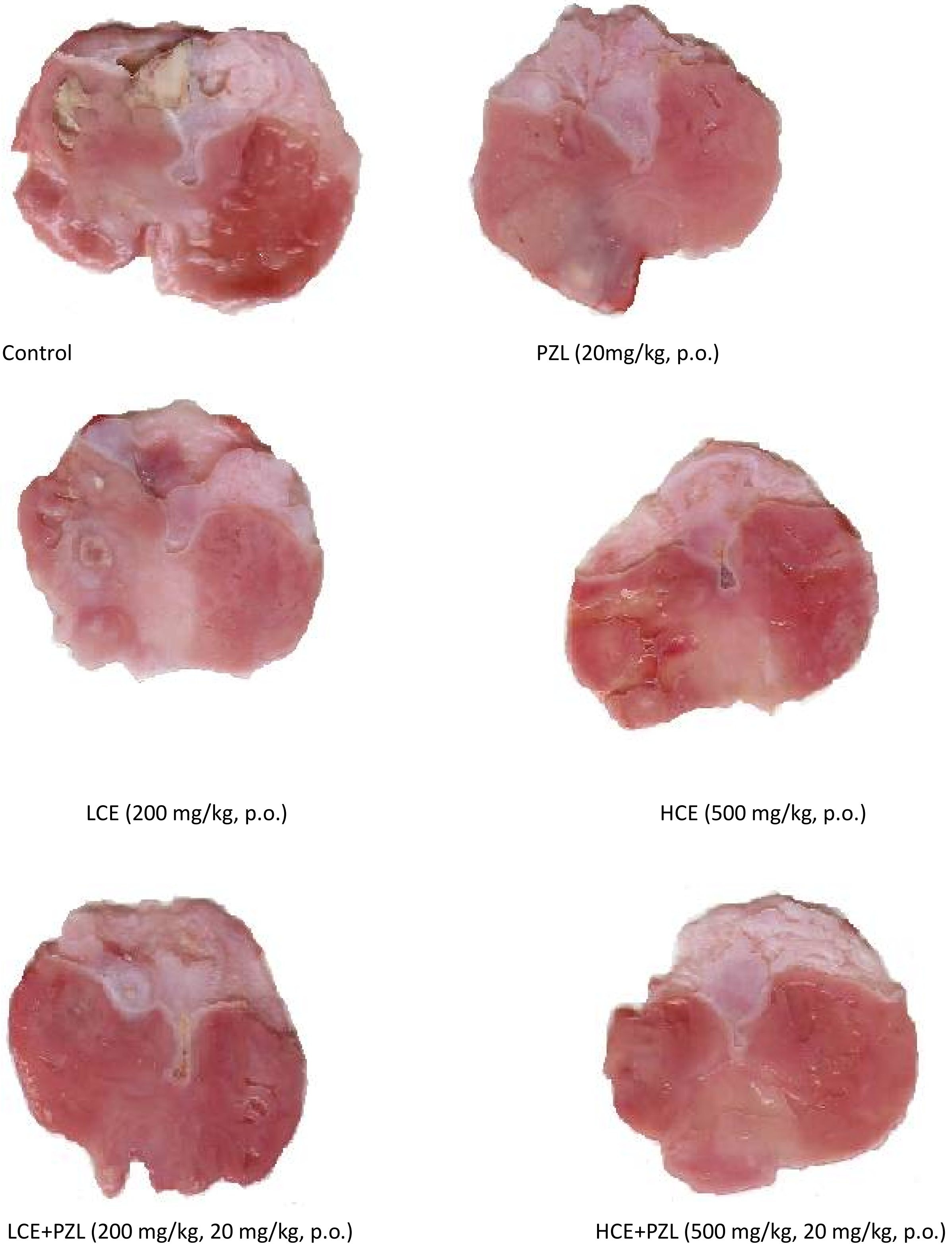

Figure S1. Photographs showing effect on ulcer healing in acetic acid induced chronic gastric ulcers.

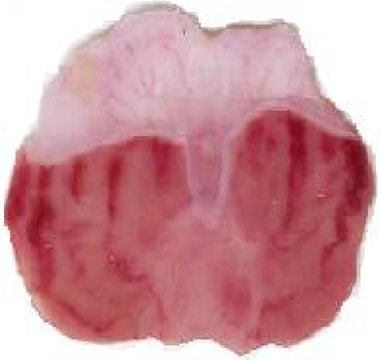

Control

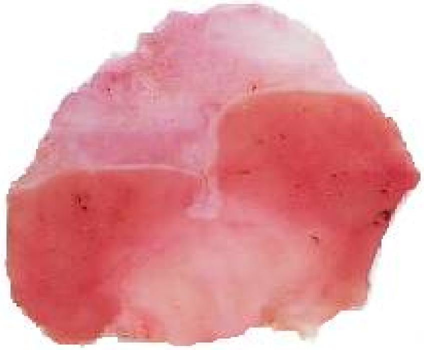

PZL (20 mg/kg, p.o.)

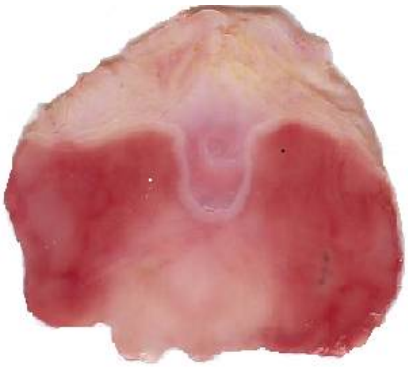

LCE (200 mg/kg, p.o.)

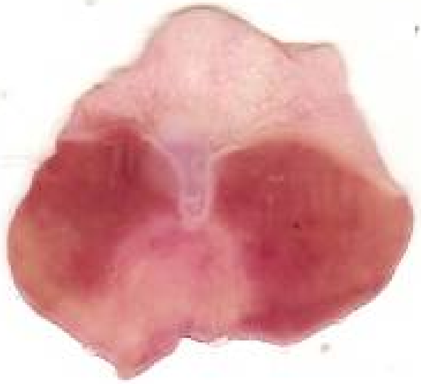

HCE (500 mg/kg, p.o.)

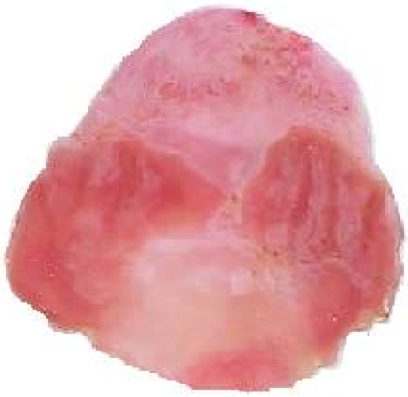

LCE+PZL (200 mg/kg, 20 mg/kg, p.o.)

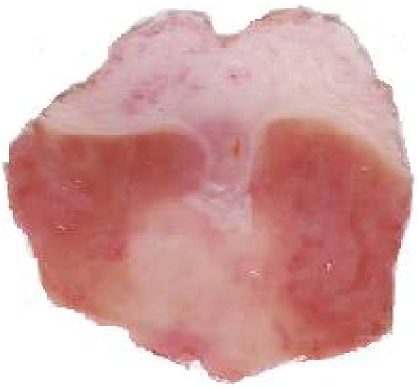

HCE+PZL (500 mg/kg, 20 mg/kg, p.o.)

Figure S2. Photographs showing effect in pyloric ligation induced gastric ulcer

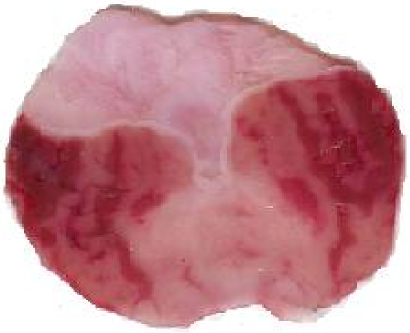

Control

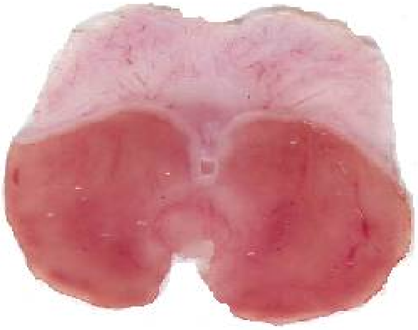

PZL (20 mg/kg, p.o.)

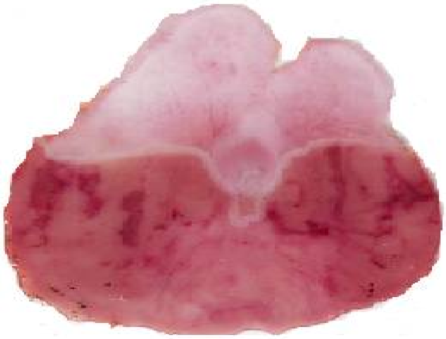

LCE (200 mg/kg, p.o.)

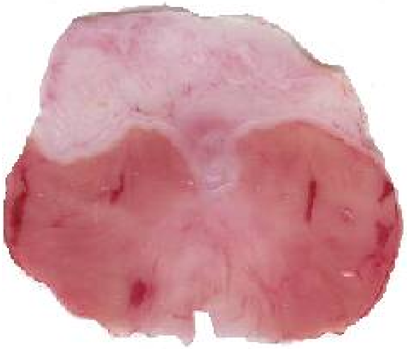

HCE (500 mg/kg, p.o.)

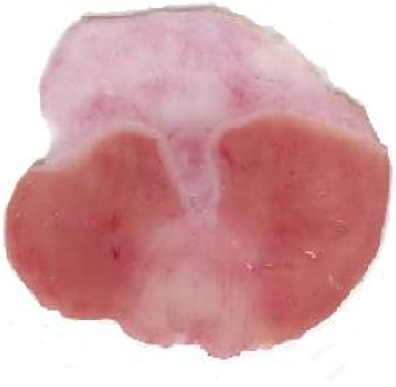

LCE+PZL (200 mg/kg, 20mg/kg, p.o.)

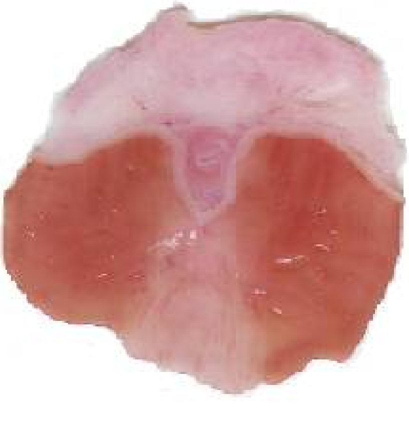

HCE+PZL (500 mg/kg, 20mg/kg, p.o.)

Figure S3. Photographs showing effect in ethanol induced gastric ulcers

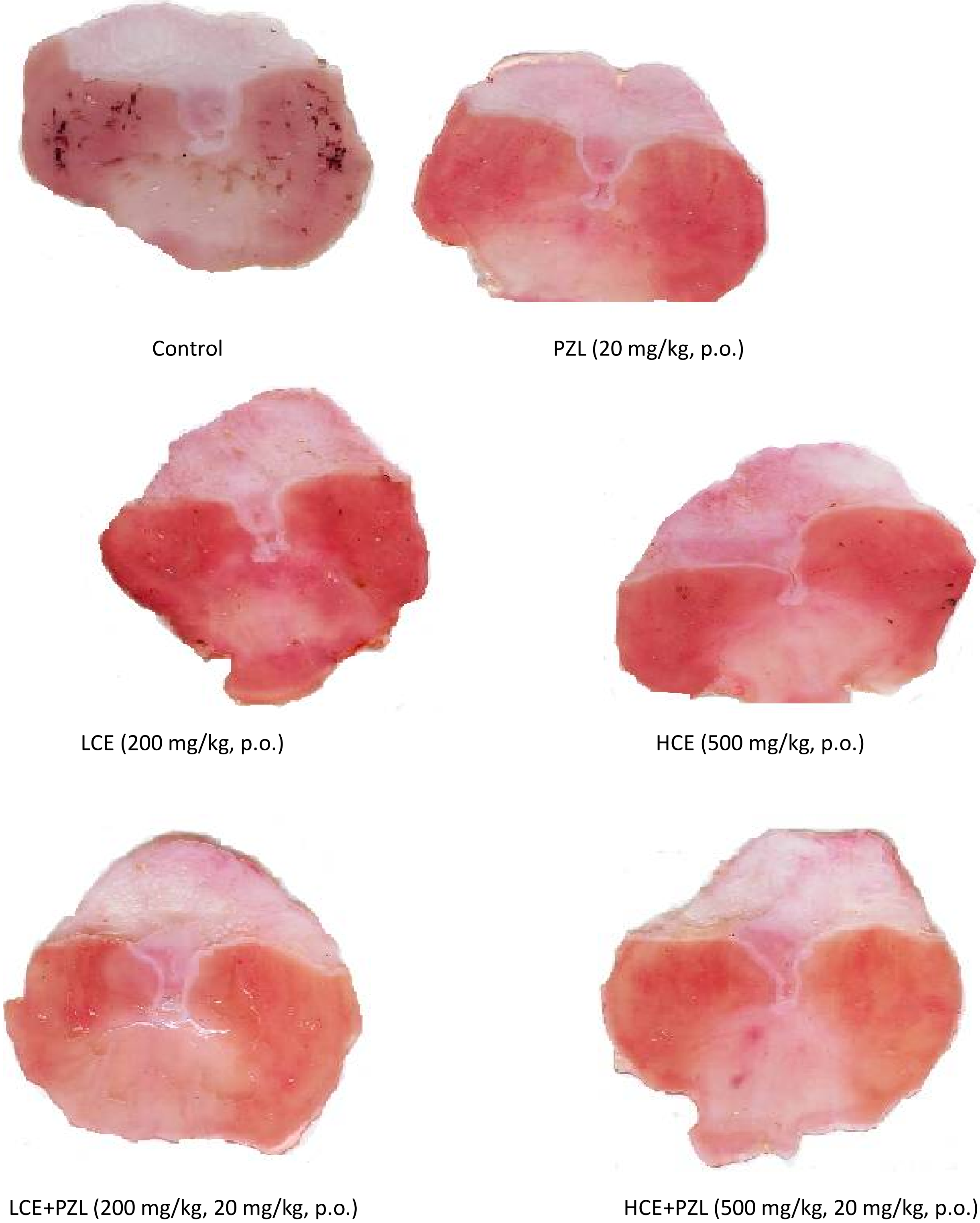

Figure S4. Photographs showing effect in stress induced gastric ulcers

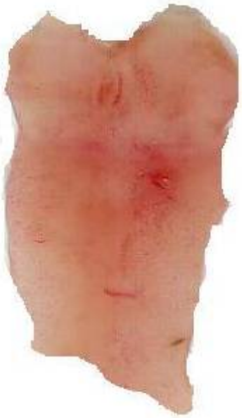

Control

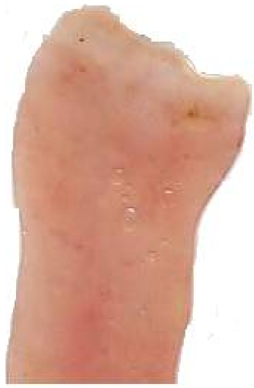

PZL (20 mg/kg, p.o.)

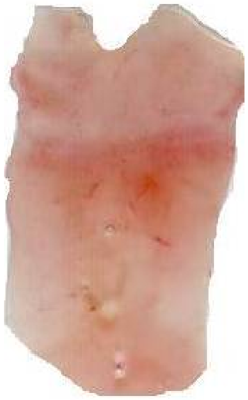

LCE (200 mg/kg, p.o.)

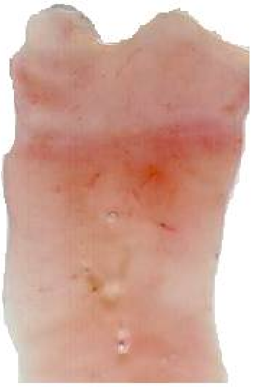

HCE 500 mg/kg, p.o.)

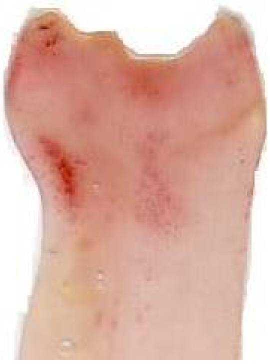

LCE+PZL

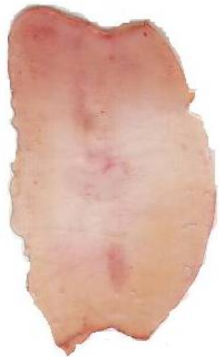

HCE+PZL

Figure S5. Photographs showing effect in cysteamine induced duodenal ulcers

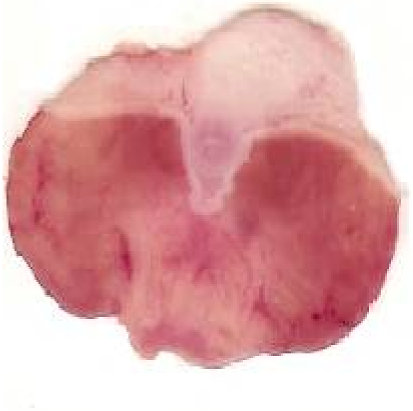

Control

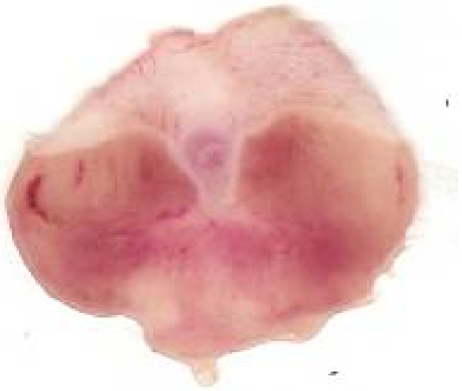

PZL (20 mg/kg, p.o.)

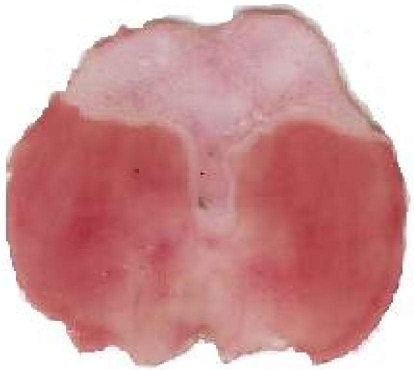

LCE (200 mg/kg, p.o.)

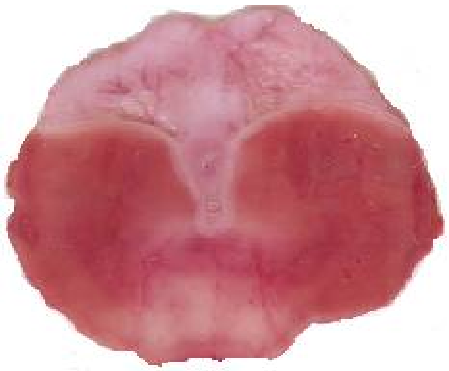

HCE (500 mg/kg, p.o.)

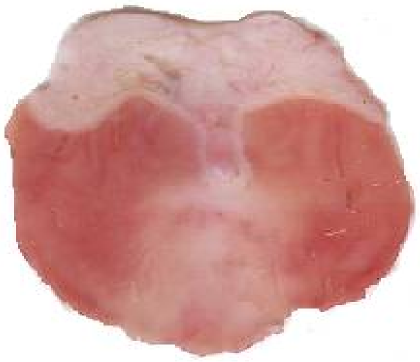

LCE+PZL (200 mg/kg, 20 mg/kg, p.o.)

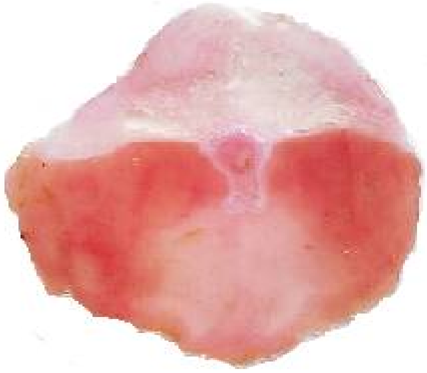

HCE+PZL (500 mg/kg, 20 mg/kg, p.o.)

Figure S6: Photographs showing effect in indomethacin induced gastric ulcers

Table S1. Phytochemical investigation for various compounds

SL NO	TEST	INFERENCE	RESULT
1. Test for alkaloids			
1.a	Hager's test	Yellow colour	+
1.b	Dragendroff's test	Orange pricipitate	+
1.c	Wagner's test	Red-brown pricipitate	+
2. Test for carbohydrates			
2a	Molish test	Violet colour	++
2b	Fehling's test	Break red colour	++
2c	Borfoed's test	Red colour	+
2d	Benedict's test	Red colour	+
3. Test for steroids, triterpenoids and glycosides			
3a	Liebermann-buchard test	Reddish- violet colour	++
3b	Salkowski test	Red colour	+
3c	Baljet test	Orange colour	++
3d	Keller killani test	Red colour	+
4. Test for saponins			
4.a	Froth test	1 cm foam	+
5 Test for tannins			
5.a	Ferric chloride test	Blue colour	+
5.b	Lead acetate test	Yellow colour	+
6 Test for proteins and Amino acids			
6.a	Millon's test	Red colour	+
6.b	Biuret test	Violet colour	+
6.c	Ninhydrin test	Violet colour	+
7 Test for flavanoids			
7.a	Ferric chloride test	Blackish red colour	+
7.b	Lead acetate test	Yellow pricipitate	+
8 Test for specific flavanoids			
8.a	Test for carotenoids	Deep blue colour	+
9	Test for phenolic compound	Radish brown colour	+

'+' represents presence of compound, '++' shows more prominence in color formation.
